# The Couple Energy & Engagement Model: a new operational theory for relationship burn-out

**DOI:** 10.3389/fpsyg.2026.1718462

**Published:** 2026-02-27

**Authors:** Benjamin Putois, Royce Anders

**Affiliations:** 1 Faculty of Psychology, Swiss Distance Learning University, Brig, Switzerland; 2 Lyon Neuroscience Research Center, National Centre for Scientific Research UMR 5292—National Institute of Health and Medical Research U1028, Bron, France; 3 EPSYLON Laboratory, Department of Psychology, University of Montpellier Paul Valéry, Montpellier, France

**Keywords:** attachment, couple burnout, couple communication, couple needs, couples, demands-resources, engagement, psychometric validation

## Abstract

Classical theories in couple psychology, such as the Vulnerability-Stress-Adaptation (VSA) model and the Systemic-Transactional Model of Dyadic Coping (STM), have substantially advanced understanding of relational dynamics by highlighting the roles of individual vulnerabilities, external stressors, and adaptive processes, particularly communication and dyadic coping. In parallel, the Job Demands-Resources (JD-R) model, originally developed in occupational psychology, demonstrates that the balance between demands and resources determines exhaustion and engagement. Although this logic has been extrapolated to intimate relationships through the notion of couple burnout, no comprehensive, operational framework has simultaneously captured both negative and positive dimensions of relational vitality. This article proposes the Couple Energy & Engagement Model (CEEM) to address these gaps. The CEEM introduces two core dimensions: couple (or relationship) energy, defined as the individual affective state reflecting vitality versus exhaustion within the relationship, and couple engagement, defined as observable behavioral investment-disengagement in the partnership. To render the CEEM falsifiable, we outline two psychometric instruments. The Couple Energy & Engagement Scale (CEES), analogous to the Oldenburg Burnout Inventory (OLBI), is designed to assess energy and engagement. The Couple Needs and Fulfillment Questionnaire (CNFQ) assesses fundamental expectations within the couple, the individual’s communication of these expectations, and the perceived partner response as resources. Together, these tools enable empirical evaluation of CEEM, factor-analytic validation, and can be used in the modeling of relational profiles via data-driven approaches, including cluster analysis, machine learning, and the Actor-Partner Interdependence Model (APIM). The CEEM thus extends existing models by offering a dynamic, ecological, and operational account of individual experience in intimate relationships, paving the way for integrative empirical research capable of measuring, validating, and theoretically refining mechanisms of relational vitality and exhaustion.

## 1 Introduction

Couple psychology has produced several major theoretical models to explain relationship satisfaction and stability. Among these, the Vulnerability-Stress-Adaptation (VSA) model (Karney and Bradbury, 1995; Ross et al., 2022) posits that relationship quality is determined by the interaction among individual vulnerabilities, external stressors, and adaptive processes, particularly communication and conflict management. The Systemic-Transactional Model of Dyadic Coping (Bodenmann, 1995, 2005) emphasizes how partners jointly face stressful situations, introducing the concept of dyadic coping. Together, these models have substantially advanced our understanding of intimate relationships by foregrounding the complexity and interdependence of contributing factors.

In parallel, work psychology has developed the Job Demands-Resources (JD-R) model (Demerouti et al., 2001, 2010), which conceptualizes exhaustion and engagement as consequences of the balance between demands and resources. This framework has been widely validated empirically and inspired robust measures such as the Oldenburg Burnout Inventory (OLBI). Some authors have transposed this logic to the relationship domain, notably via the construct of marital or couple burnout (Pines, 1996). In this context, burnout in intimate relationships can be defined as a state of emotional exhaustion and progressive disengagement that emerges when relational demands persistently exceed the resources available to meet them. This state is characterized by feelings of depletion, reduced emotional availability, and a diminished sense of involvement in the relationship, without necessarily implying relationship dissolution. However, these approaches remain focused on negative endpoints and do not adequately capture the positive dimensions of relational vitality or offer a continuous, falsifiable metric. Within the Job Demands-Resources (JD-R) framework, burnout represents one pole of a broader continuum that also includes positive states such as engagement and vitality. Applied to intimate relationships, relationship vitality refers to an affective state of emotional energy and relational aliveness supported by sufficient resources. Introducing vitality alongside burnout is therefore theoretically consistent with the JD-R logic and provides the conceptual basis for the Couple Energy & Engagement Model (CEEM).

Against this backdrop, the present article introduces the CEEM as an integrative theoretical framework for studying relational functioning. Rather than focusing solely on relationship outcomes such as satisfaction or stability, the CEEM draws on interdisciplinary perspectives from relationship science and the JD-R model to examine how relational demands and resources interact within intimate partnerships. By adopting this perspective, the model aims to capture a broader and more differentiated spectrum of relational functioning, encompassing both negative and positive experiences, and to provide a coherent foundation for future empirical investigation of relational burnout, vitality, and engagement. In this sense, the CEEM can be understood as a “zoom-in” model that complements existing frameworks by focusing on more proximal affective states and observable behaviors within intimate relationships. Rather than replacing broader models of relationship functioning, the CEEM refines them by examining how relational demands and resources are experienced and enacted at the individual level through energy and engagement. These dimensions are shaped by the interactions between demands (fundamental needs inspired by Panksepp’s primary emotional systems), resources (intrapersonal, dyadic, and extradyadic), and communication, which functions as the central mediator. Intimate relationships represent a central context of adult life, with profound implications for emotional regulation, mental health, and overall quality of life. When relational demands chronically exceed available resources, individuals may experience relational exhaustion or disengagement, with consequences extending beyond the couple itself. Conversely, intimate partnerships can also function as major sources of vitality and support. Studying both relational burnout and vitality therefore allows relationship science to capture how relationships may either undermine or enhance individual well-being.

## 2 Theoretical framework of the Couple Energy & Engagement Model (CEEM)

The JD-R model was originally developed to explain burnout and engagement in occupational contexts. Within this framework, demands refer to aspects of the environment or role conditions that require sustained physical, emotional, or cognitive effort, such as workload, time pressure, role ambiguity, or interpersonal conflict. Importantly, demands are considered neutral in themselves and become detrimental primarily when they are excessive or when adequate resources to meet them are lacking. Resources, in contrast, are defined as factors that help individuals meet demands, reduce associated costs, or foster motivation and engagement. These resources may originate from different sources, including the individual (e.g., skills, emotion regulation capacities), the interpersonal context (e.g., social support), or the broader environment (e.g., autonomy, material or contextual support). In adapting the JD-R framework to intimate relationships, the CEEM deliberately adopts a zoom-in perspective. Rather than focusing primarily on external or contextual demands (e.g., work stress, financial strain, caregiving burden), which are already well documented in relationship research, the CEEM conceptualizes demands at a more proximal level as internally experienced relational needs. This choice is theoretically justified by the model’s focus on how the relationship is lived and regulated from within, through affective states and behavioral investment. In the CEEM, contextual stressors are not excluded; instead, they are understood as distal or background demands that shape relational functioning indirectly, by intensifying needs, constraining available resources, or impairing communication. By centering demands on needs, the CEEM maps the JD-R logic onto the core regulatory functions of intimate relationships while remaining compatible with broader contextual models. Within the CEEM, the conceptual equivalence between relational needs and couple demands is intentionally defined at two complementary levels. At a proximal level, demands primarily refer to one partner’s relational needs and expectations as they are experienced internally and potentially placed on the partner through communication and behavior. At a more distal level, demands also encompass the broader conditions under which the relationship is conducted, such as contextual stressors or life constraints, which may shape or intensify relational needs without being directly addressed to the partner. The CEEM prioritizes the proximal level in order to capture how demands are subjectively lived and regulated within the relationship, while remaining theoretically compatible with broader contextual conceptions of demands derived from the JD-R framework. The CEEM rests on three pillars: (i) fundamental needs as couple (partner) demands, and their fulfillment being linked to couple vitality; (ii) the multiple resources available to meet them, or lack thereof (source of stress, couple disengagement), and (iii), communication as the central mediator.

The CEEM therefore assumes that fundamental needs constitute couple demands. As described by Panksepp (1998) in his primary emotional systems model, these include the need for exploration (SEEKING), attachment and safety (CARE), play and laughter (PLAY), sexuality (LUST), as well as the need to regulate fear (FEAR), anger (RAGE), and distress (PANIC/GRIEF). In intimate partnerships, such needs translate into concrete expectations: receiving affective support, sharing joyful moments, being encouraged in one’s projects, experiencing fulfilling sexual intimacy, and being able to express negative emotions without fear of rejection. These demands are universal, yet their intensity and modes of expression vary across individuals as a function of attachment history, personality, and life trajectory. They form the foundation upon which relational equilibrium rests. That is, sufficient fulfillment of each partner’s needs supports a couple’s vitality, or energy.

Resources mobilized to meet these needs can be of three types. Intrapersonal resources include emotion regulation capacities, self-esteem, resilience, physical health, and spirituality. These enable individuals to meet needs without exclusive dependence on the partner. Extradyadic resources encompass social support, professional recognition, and cultural or community activities. This distinction introduces an ecological perspective and helps explain why some individuals maintain vitality despite an unsatisfying relationship, whereas others become depleted when exclusively dependent on the partner. Finally, couple resources are provided by the partner-support, validation, encouragement, and affective security. Given the unique proximity and salience of intimate bonds relative to other human relationships, these resources are privileged in regulating needs. The distinction between intrapersonal, dyadic, and extradyadic resources is grounded in an ecological view of intimate relationships (Michael and Ben-Zur, 2024). This categorization reflects the fact that relational functioning depends on resources located within the individual, provided by the partner within the attachment bond, and originating from the broader social context. It is particularly appropriate for couple research, as it captures both the privileged regulatory role of the partner and the embeddedness of intimate relationships within wider personal and social systems (Feeney and Collins, 2015). This distinction introduces an ecological perspective and helps explain why some individuals may maintain a sense of personal vitality or subjective energy despite experiencing an unsatisfying intimate relationship, due to the availability of intrapersonal or extradyadic resources. In the CEEM, a perceived lack of resources to meet a partner’s perceived needs is expected to generate stress at the individual level for the partner who experiences insufficient capacity to respond, which may subsequently reduce behavioral availability and engagement; through interaction and communication processes, these changes may also affect the partner’s engagement over time. Consistent with the JD- R framework, the CEEM assumes that relational resources may both buffer the negative effects of high relational demands on stress and disengagement, and exert a direct motivational influence by supporting vitality and adaptive engagement when resources are sufficient, even under demanding relational conditions.

Communication is the central mediator in the CEEM. It transforms demand into practically accessible resources. Clear, empathic, and validating communication promotes congruence between demands and couple resources. Conversely, deficient communication can create gaps between expectations and partner provisions, heightening the risk of boredom, disengagement, or exhaustion. This emphasis aligns with VSA, yet CEEM goes further by linking communication not only to global marital satisfaction but directly to energy and engagement. This demands-resources articulation is also consonant with the physiological stress tradition in Selye’s work, wherein adaptive responses depend on the ratio between environmental demands and the organism’s adaptive capacity (Selye, 1956).

## 3 Dimensions of the CEEM: energy and engagement

The CEEM introduces two central dimensions: couple energy and couple engagement.

Couple energy (or vitality) is defined as the individual affective state associated with the relationship. It lies on a continuum from exhaustion (equivalent to couple burnout), boredom (characterized by under-stimulation) and affective paucity, through equilibrium (satisfaction), to vitality (heightened emotional energy and relational dynamism). Unlike approaches focused solely on exhaustion, the CEEM proposes a dynamic continuum that captures both negative and positive fluctuations in relational experience. In the CEEM, the energy dimension is defined at the individual level and explicitly reflects the perceived fit between one’s own relational needs or demands, and the resources one perceives as available in the couple to meet them; particularly, those provided by the partner. Thus, the focal process primarily concerns perceptions of “my needs versus my partner’s resources to satisfy them,” as experienced by the individual.

Couple engagement refers to observable behaviors of investment in the relationship. It spans a continuum from disengagement (avoidance and withdrawal), depersonalization, marked by indifference and coldness, to balanced engagement (constructive involvement), and then to over-engagement, characterized by fusion, dependence, and excessive sacrifice. In the CEEM, the engagement dimension can be defined at the individual level and explicitly reflects an individual’s perceptions of their capacity to sufficiently meet their partner’s needs. Thus, the focal process primarily concerns perceptions of “the resources I can draw upon in order to respond to my partner’s demands.” An individual perceiving low or insufficient resources to meet their partner’s needs (arising from exhaustion, feeling incompatible, or powerless to please the other for example), would be associated with states toward disengagement or distancing. In contrast, an individual perceiving an adequate capacity, resources, or ease to meet their partner’s needs (and, e.g., perceived gains), would be associated or reinforcing with states of couple engagement. Finally, if an individual provides resources in excess of what is needed, or, perceives needing monumental efforts to satisfactorily meet their partner’s demands, they would be considered over-engaged, which over time, would lean towards exhaustion (and may be likely to ultimately trigger in disengagement).

**Figure 1:**
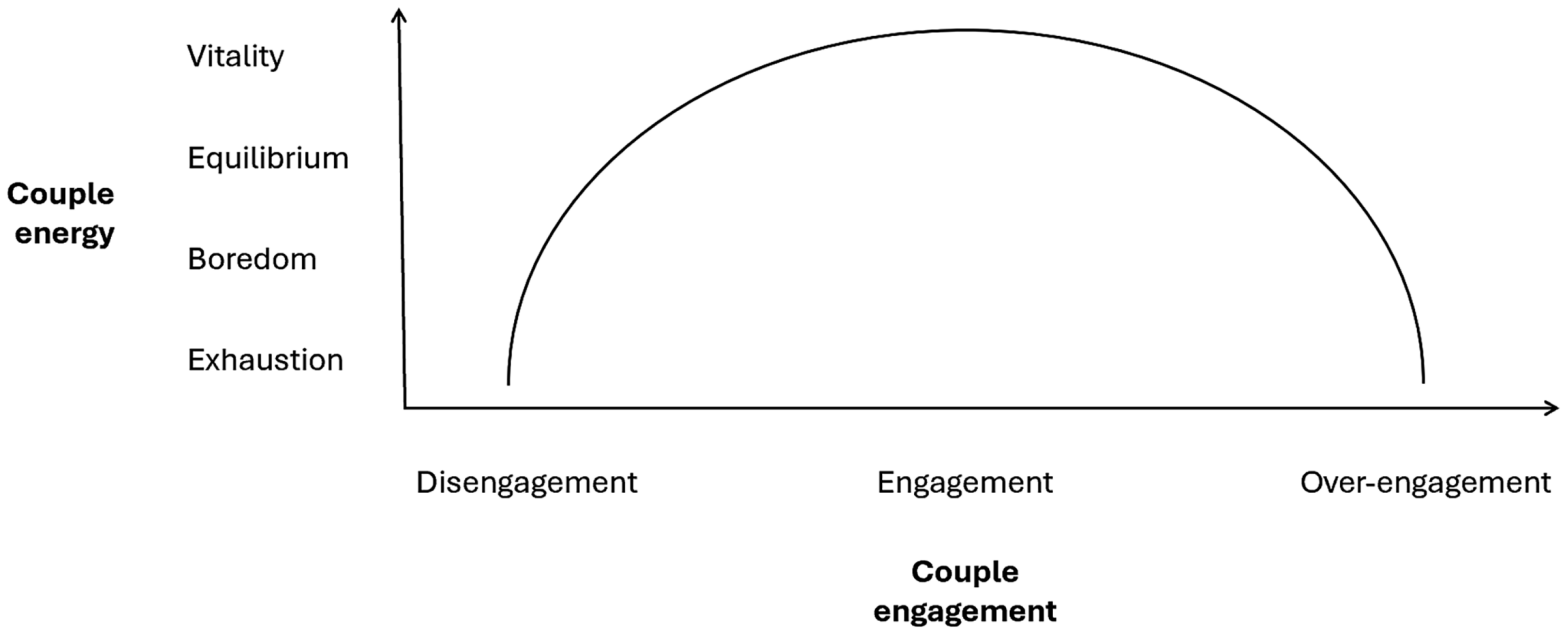
Hypothesized link between couple energy and couple engagement.

Therefore, as visualized in Figure 1, we can hypothesize that the link between couple energy and couple engagement would follow a dynamic akin to an inverted-U function, similar to the Yerkes and Dodson (1908) law. For example, low levels of engagement (e.g., depersonalization, disengagement) would be natural, or a commonplace protective response in cases of low vitality (e.g., personal exhaustion, boredom, or lack of stimulation with the couple). In contrast, balanced engagement (i.e., constructive implication) would be associated in like with good relationship vitality (e.g., couple satisfaction, energy). That is, where the resources-demands link is appropriately balanced (e.g., perceived ease in meeting needs, or compensation of “spent” resources with incoming needs being met). And finally, over-engagement representing an excess of resources for meeting (or surpassing) demands, would be associated with reduced vitality (e.g., over time exhaustion, or alternatively, boredom such as in the daily routine). The temporal dynamics are therefore important to take into account to recognize or understand how this inverted-U trend would manifest. For example, early in the relationship, increasing levels of engagement, towards over-investment, might initially enhance vitality (e.g., “love bombing”), but over time, without adjustment from the partner, energy could collapse. This is why burnout, as we have defined it, can only be assessed as resulting from a prolonged imbalance between relational demands and available resources. The symmetry or asymmetry of demands and resources between partners should therefore be considered in couples who have been together for a sufficient amount of time when testing this hypothesis.

Within the broader field of relationship research, couple energy and couple engagement are related to, but distinct from, established constructs such as relationship satisfaction, commitment, and attachment security. Whereas relationship satisfaction primarily reflects a global evaluative judgment, couple energy captures the individual’s affective experience of the relationship, ranging from exhaustion and boredom to vitality. Similarly, couple engagement overlaps with commitment but focuses more specifically on observable patterns of behavioral involvement rather than on motivational intention alone. Attachment security is not conceptualized as an outcome in the CEEM, but rather as a distal factor shaping needs, communication, and access to relational resources. In this way, energy and engagement are positioned as proximal experiential and behavioral dimensions within the broader nomological network of relationship functioning.

In analogy with the JD-R model, the CEEM posits that each partner has fundamental needs or demands (involving cognitive and physical aspects) that link to core emotions, and that their partner can serve as a resource for meeting those needs, thereby influencing the level of need fulfillment experienced in the relationship. Within CEEM, two analytically distinct dimensions are distinguished: The first dimension focuses on the individual’s own fundamental needs within the relationship and their ability to communicate them effectively. The second dimension concerns the individual’s perception of their partner’s efforts to meet those needs. Conversely, each person also perceives fundamental needs in their partner, and overt demands, and attempts to respond to them. From this perspective, the couple functions as a co-regulating system for fundamental needs, highlighting especially those of emotional nature. In the CEEM, the energy dimension is explicitly conceptualized as reflecting the perceived fit between an individual’s relational needs or demands and the resources available to meet them, particularly those provided by the partner. When individuals perceive that their core needs are adequately met by available relational resources, energy is expected to increase; conversely, perceived mismatches between needs and resources are expected to be associated with lower energy, exhaustion, or boredom.

In the CEEM, fundamental need fulfillment is modeled as a central predictor of the couple energy dimension (boredom-satisfaction-vitality); and partner perception, as possessing the resources to adequately fulfill a partner’s fundamental needs he/she perceives (and overt demands), as a central predictor of the couple engagement dimension (depersonalization-disengagement-engagement), which may also relate to stress level.

## 4 Comparison with existing models

The CEEM extends existing frameworks while offering several innovations. Relative to couple burnout (Pines, 1996; Pines and Renato, 2003), CEEM moves beyond a strictly exhaustion-centered view by proposing a dynamic continuum that also includes boredom, satisfaction, and vitality. This perspective enables assessment not only of negative outcomes but also of states of relational dynamism.

Compared with the JD-R model (Demerouti et al., 2001), the CEEM transposes the demands-resources logic to intimate relationships but adds communication as a central mediator and integrates an ecology of resources. Whereas the JD-R model focuses solely on exhaustion and disengagement, the CEEM proposes a broader framework that follows a continuum: from emotional exhaustion to vitality, and from relational disengagement to marital engagement. In addition to its strain pathway, whereby excessive demands lead to stress and burnout, the model also includes a well-established motivational pathway in which resources directly foster positive outcomes such as engagement. Moreover, the JD-R framework explicitly posits a buffering hypothesis, according to which resources can attenuate the negative effects of high demands on strain. These core assumptions provide an important theoretical foundation for CEEM. In line with earlier sections, vitality in CEEM refers to an individual affective state of energy and aliveness experienced by the person within the relationship, rather than to a property of the relationship itself. In this sense, vitality represents the positive pole of the individual energy dimension and is conceptually aligned with the motivational pathway described in the JD-R framework.

Relative to the VSA model (Karney and Bradbury, 1995; Ross et al., 2022), CEEM does not seek to replace established non-operational outcomes such as relationship quality or stability, which have been extensively operationalized and validated in the literature. Rather, CEEM shifts the analytical focus toward the individual-level affective and behavioral experience of the relationship, conceptualized through the dimensions of couple energy and couple engagement. Whereas VSA primarily models how vulnerabilities, stressors, and adaptive processes shape global relationship outcomes over time, CEEM complements this approach by offering fine-grained, falsifiable indicators of how the relationship impacts each partner’s vitality and behavioral investment at any given point. In this sense, CEEM can be viewed as an operational extension of VSA, providing proximal experiential metrics that may help explain variations in relationship quality and stability observed in longitudinal models.

Compared with the Systemic-Transactional Model of Dyadic Coping (Bodenmann, 1995, 2005), the CEEM does not differ primarily in the acknowledgment of external stressors, which are already central to the systemic-transactional framework. Rather, the distinction lies in the level of analysis and in the nature of the modeled outcomes. While the Systemic-Transactional Model focuses on how partners jointly cope with stressors and how dyadic coping processes influence relationship functioning, CEEM shifts the focus toward the individual-level affective and behavioral consequences of the relationship itself, operationalized through the dimensions of couple energy and couple engagement. Moreover, the CEEM explicitly conceptualizes intrapersonal, dyadic, and extradyadic resources within a unified demands-resources framework, and places communication as a mediator linking needs, resources, and experiential outcomes. From a theoretical standpoint, the CEEM thus formulates the proposition that individual vitality within the relationship may be sustained or depleted depending on the configuration of available resources, even under comparable levels of dyadic coping. These propositions remain to be empirically tested and are intended to guide future data-driven and dyadic research rather than to assert causal explanations at this stage.

## 5 The CEEM as a falsifiable model: development of psychometric instruments

For the sake of conceptual clarity and falsifiability, CEEM relies on a consistent set of terms to describe affective states and outcomes. Throughout the manuscript, the term energy refers to the core individual affective dimension, ranging from exhaustion to vitality. Within this continuum, exhaustion denotes the negative pole, vitality the positive pole, and equilibrium is used to describe an intermediate state characterized by relative stability and functional regulation. Relationship satisfaction is not treated as a distinct outcome in CEEM, but as an empirical indicator typically associated with this equilibrium state. These terms are used consistently across sections to ensure that each construct refers to a unique and identifiable position within the model.

Several instruments in the literature can be used to approximate certain CEEM dimensions, yet none of them are able to cover the model in its entirety. For example, one may note the Brief Accessibility, Responsiveness, and Engagement Scale (Sandberg et al., 2012) that assesses accessibility and engagement in the relationship; the Mutuality and the Marital Engagement -Type of Union Scale (Singer et al., 2015) evaluates mutuality and marital engagement; and the Self-Expansion Questionnaire (Aron et al., 1992) captures relational vitality via self-expansion attributed to the partner. These instruments are pertinent but do not clearly differentiate, as CEEM does, that is, between couple energy (affective state: exhaustion, boredom, equilibrium, vitality) and couple engagement (behavioral investment: depersonalization, disengagement, balanced engagement, over-engagement).

The causal structure assumed in CEEM is consistent with the JD-R framework and incorporates both direct and interactive pathways. Relational needs or demands may exert a direct influence on individual emotional, arousal-related, and behavioral states, particularly when they are chronically intense or salient. In parallel, CEEM explicitly posits an interaction (fit) process, whereby the impact of needs on energy depends on the availability of resources to meet them, with communication functioning as a central mediating mechanism. The measurement strategy places particular emphasis on this fit logic, as it provides a concrete and falsifiable means of operationalizing both strain-related and motivational pathways within a unified framework. More recently, instruments explicitly grounded in the JD-R model framework have been proposed for the relational domain. Notably, the Antecedents of Relationship Burnout Scale (ARBS) (Thompson et al., 2025) was developed to assess relational demands and resources associated with couple burnout. This scale represents an important advance by formally transposing the JD-R logic to intimate relationships and by focusing on predictors of relational exhaustion. However, its primary scope remains centered on antecedents of burnout and does not encompass positive or non-pathological relational states. In contrast, CEEM extends this framework by proposing a continuous model that integrates not only exhaustion, but also boredom, equilibrium, vitality, and engagement, thereby capturing the full spectrum of individual affective and behavioral experiences within the relationship.

In respect to validated measures pertaining to the needs-communication-resources triad, several tools provide partial coverage. The Scale of Self-perceived Communication in the Couple Relationship (Iglesias García et al., 2019) assesses perceived communication between partners, while ENRICH (Fowers and Olson, 1989) examines diverse facets of relationship quality, including communication, expectations, and satisfaction. The Central Relationship Questionnaire (Weinryb et al., 2000) and the Basic Psychological Needs Scale (Deci and Ryan, 2000) address central relational expectations and fundamental emotional needs, respectively. However, none of these instruments articulate, within a single framework, specific couple/partner needs, their effective communication, and the perceived partner response as resources.

Accordingly, despite their value, these questionnaires do not provide an integrated measurement of relational experience as proposed by the CEEM. This justifies developing two complementary tools specifically designed to test the model:

The first is the Couple Energy & Engagement Scale (CEES), inspired by the OLBI (Demerouti et al., 2003). It would comprise two subscales: one measuring energy (exhaustion, boredom, satisfaction, vitality) and one measuring engagement (depersonalization, disengagement, balanced engagement, over-engagement). Items would capture both subjective affective states and observable behaviors.

The second instrument is the Couple Needs and Fulfillment Questionnaire (CNFQ), developed to assess three interrelated dimensions of intimate relationships. The first dimension pertains to demands, and evaluates how individuals represent their own and their partner’s fundamental needs within the couple. These needs are conceptualized in alignment with Panksepp’s seven primary emotional systems that incorporate also cognitive (e.g., trust, autonomy, reassurance) and physical (e.g., lust) needs among the emotional. For example, emotional and financial security, as well as the need for reassurance, are understood as part of the regulation of fear. Needs related to trust and fidelity help regulate grief, while the capacity to express anger and frustration relates to the regulation of rage. Autonomy, independence, and encouragement are linked to seeking, while affection, tenderness, and love correspond to the care system. Shared humor, playfulness, and a sense of complicity reflect the regulation of play, and finally, sexuality, fantasies, and shared desires pertain to the regulation of lust. The second dimension addresses the couple’s ability to communicate these needs, emphasizing the clarity and effectiveness with which each partner expresses their emotional demands within the relationship. The third dimension, fulfillment, focuses on resources, referring to the individual’s perception of how well their partner fulfills their needs, as well as how effectively they themselves meet their partner’s needs. This questionnaire is self-report and can be completed by both partners.

The interaction among the subdimensions of the CFNQ is expected to correlate with the CEES. For instance, when an individual considers a particular need, such as the need for care, as highly important, and perceives their partner as meeting it, the CEES should reflect a positive emotional valence score. Conversely, when such a need is expressed but remains unmet, the CEES is expected to show a negative valence. Furthermore, when a partner feels they invest significant effort in meeting the other’s needs but perceives little reciprocity, this asymmetry may lead to increased emotional exhaustion. The CNFQ also provides insight into how the distribution of fulfilled needs influences relational dynamics. A balanced fulfillment of needs is hypothesized to predict greater marital satisfaction and longer relationship duration. In contrast, a consistently low level of need fulfillment may lead to progressive disengagement and a shorter lifespan of the relationship. Partial fulfillment of needs might be associated with ambivalence, where one partner hesitates to leave but chooses to stay nonetheless. Importantly, the model also accounts for changes over time. In long-term relationships where some needs are fulfilled while others are neglected, the frequency of need expression may decline due to negative reinforcement, leading to a gradual reduction in relational vitality and engagement. Together, the CEES and the CNFQ offer a comprehensive framework for the scientific investigation of couple burnout, while also serving as a clinically valuable tool for couple therapy and intervention.

## 6 Proposed methodology

Empirical validation of the CEEM is projected to proceed in three phases. First, the CEES and CNFQ are planned to undergo rigorous psychometric validation, including exploratory and confirmatory factor analyses, internal consistency estimation, and assessment of convergent and discriminant validity. The CEES and CNFQ are expected to correlate with several established measures (in addition to the previously mentioned questionnaires), including the Sociosexual Orientation Inventory (Penke and Asendorpf, 2008), the Dyadic Adjustment Scale (Spanier, 1976), the Physical Attraction Scale (Karandashev et al., 2020), the Passionate Love Scale (Hatfield and Rapson, 1990) and the Romantic Love Scale (Rubin, 1970).

In the second phase, data-driven analyses will be conducted to comprehensively explore the pertinence of the selected instruments and measures, and more thoroughly explore relational patterns within the CEEM framework. That is, two main approaches of data-driven analyses, each with distinct objectives, can be noted. First, given the thorough spectrum of variables that will assessed, implying a need for managing the high dimensionality of the data sets, predictive models recognized for robust *feature selection* (e.g., Elastic Net, Lasso Regression) will be used to identify the variables that are the most associated with the centrally thematic variables of the CEEM (e.g., couple engagement, energy). Furthermore, these approaches will be used to quantify the strength of association of these variables, whilst others are accounted for (ecological pertinence), and to also verify said results as a function of the overall predictive performance of the model, compared to other model candidates. These powerful linear modeling approaches, classically associated with the label: supervised learning, however, assume one unique set of weights for all participants and are hence not designed to detect population subgroups (e.g., profiles, classes, clusters) that may present distinct properties or even exceptional trends. This brings to light the second main data-driven approach that will be applied, namely *latent profile analysis* (assuming a multivariate normal distribution if adequate) or otherwise *clustering* (based on a distance measure), which are associated with the term: unsupervised learning. Specifically, this second approach of analyses will be used to identify distinct relational profiles across the CEES and CNFQ dimensions. For example, profiles may emerge that combine high couple energy with low engagement, or vice versa, revealing asymmetries in emotional states and behavioral investment within couples. These profiles can then be mapped onto empirically observed relational trajectories (e.g., stability, burnout, ambivalence, separation), providing a dynamic understanding of how specific configurations of energy, engagement, and need fulfillment relate to long-term relationship outcomes.

In the third phase, the CEEM will be tested in dyadic contexts using the Actor-Partner Interdependence Model (APIM) (Kenny et al., 2006). This statistical framework enables the simultaneous analysis of both actor effects (i.e., how an individual’s relationship energy, engagement, or need fulfillment influences their own relational outcomes) and partner effects (i.e., how these same variables affect their partner’s outcomes). The APIM is particularly suited to examining the interdependence between partners, and will allow researchers to assess whether, for example, one partner’s emotional exhaustion or unmet needs contribute to decreases in the other’s engagement or satisfaction. Through dyadic modeling, CEEM can be validated not only as a theory of individual relational experience, but also as a framework for understanding couple-level dynamics and reciprocal emotional regulation within intimate partnerships.

Although CEEM is conceptualized as a dynamic model, its temporal dimension can be examined using several complementary empirical approaches. First, longitudinal study designs allow for the assessment of within-individual and within-dyad changes in couple energy and engagement over time. Growth-based models (e.g., latent growth curve modeling) can be used to examine trajectories of relational vitality or exhaustion, while latent transition or latent change models may capture shifts between relational profiles identified through data-driven analyses. Second, repeated-measures designs enable the investigation of short-term fluctuations in energy and engagement as a function of changing demands, resources, or communication patterns. Finally, the APIM can be extended longitudinally to examine how changes in one partner’s needs, resources, or relational energy predict subsequent changes in the other partner’s outcomes. Together, these approaches provide a coherent methodological framework for testing the dynamic assumptions of CEEM across multiple time scales.

Importantly, dyadic analyses also allow for the examination of actor-partner asymmetries, such as discrepancies between an individual’s perceived investment in the relationship and their perception of the partner’s responsiveness or resource provision. These asymmetries may represent particularly informative configurations within the CEEM framework, as they are theoretically expected to relate to emotional exhaustion, disengagement, or over-engagement. Explicitly modeling such mismatches constitutes a central contribution of CEEM to dyadic research and will be a key focus of future empirical testing.

## 7 Discussion

The CEEM offers several theoretical and empirical contributions. Theoretically, it introduces two operational dimensions - energy and engagement—that directly measure the relationship’s impact on the individual. It advances an ecological approach integrating the full spectrum of available resources and places communication at the core of relational dynamics. Empirically, it is amenable to rigorous falsification through appropriate psychometric instruments and data-driven methods (the latter, reducing confirmation bias). From a clinical perspective, CEEM may offer a promising framework for future applications, contingent upon empirical validation of its core constructs and instruments. If supported empirically, the model could inform interventions aimed at improving communication, strengthening intrapersonal and extradyadic resources, and regulating the balance between demands and resources within couples. By distinguishing between affective states (energy) and behavioral investment (engagement), CEEM has the potential to help clinicians formulate more precise hypotheses regarding relational difficulties and to tailor interventions accordingly. At this stage, these clinical implications should be considered as theoretical extensions of the model rather than established clinical recommendations.

Beyond these immediate contributions, a data-driven CEEM also opens the path toward the establishment of a more comprehensive general model of romantic partnerships, or couples. In such a framework, the fundamental needs would be moderated by a series of individual and developmental variables, including adverse childhood experiences (Bernstein et al., 2011), attachment style (Brennan et al., 1998), history of intimate partner violence (Straus et al., 1996), emotion regulation capacities (Kaufman et al., 2023), complex PTSD (Cloitre et al., 2013), structural dissociation of the personality (Hart et al., 2007), tendencies toward self-criticism and self-reassurance (Gilbert et al., 2004), and broader motivational frameworks such as the Psychological Needs Scale (Deci and Ryan, 2000). Communication, already conceptualized as the central mediator in CEEM, could be further refined to include dyadic coping (Bodenmann, 2005), contextual stress exposure, interpersonal empathy (Interpersonal Reactivity Index; Davis, 1980), and general coping styles (Carver et al., 1989). Similarly, the perceived partner responses could be enriched by constructs such as physical and emotional attraction (Aron et al., 1992; Yela, 2006), romantic love (Hatfield and Sprecher, 1986), and global relationship satisfaction (Funk and Rogge, 2007).

The outcomes of such a general model would extend beyond couple energy and engagement to include interpersonal dependency (Hirschfeld et al., 1977), intimate partner violence (Straus et al., 1996), as well as individual mental and physical health indicators. Critically, this general model must be tested at the dyadic level using the APIM (Kenny et al., 2006) enabling the simultaneous assessment of actor and partner effects, as well as the covariance between partners’ residuals.

The potential impact of this broader project is twofold. Scientifically, the project would establish the foundations of a new paradigm in relationship and attachment research. It would generate unprecedented large-scale data allowing for the empirical validation of hypotheses that have remained largely theoretical: for example, the co-activation of attachment styles, destructive cycles in passionate relationships, and the role of dissociation and trauma. Such work would help answer central questions: Why do certain passionate couples self-destruct? Which attachment matchings are genuinely protective? What is the impact of love on mental, physical, and sexual health? Clinically, the project would yield prevention and assessment tools enabling clinicians, couple therapists, and social workers to identify at-risk profiles and support functional couples. It would allow for the identification of higher probability “compatible matchings” that foster stability and well-being, while also inspiring new therapeutic approaches grounded in attachment theory and emotion regulation. Clinical implications also include the development of concrete recommendations to disrupt the intergenerational transmission of violence.

### 7.1 Limitations and future directions

Several limitations of the present work deserve consideration. First, as a theoretical model, CEEM inevitably shares conceptual ground with existing constructs in couple and relationship research, including relationship satisfaction, commitment, engagement, dyadic coping, and burnout. While the model aims to integrate and reorganize these notions within a unified demands-resources framework centered on energy and engagement, its added value will ultimately need to be demonstrated empirically. In particular, future studies will be required to assess whether CEEM explains variance in relational functioning beyond that captured by well-established measures.

Second, the operationalization of fundamental needs within intimate relationships raises both conceptual and methodological challenges. Although anchoring these needs in Panksepp’s primary emotional systems provides a coherent theoretical foundation, translating complex emotional, cognitive, and interpersonal processes into self-report instruments necessarily involves a degree of abstraction and simplification. Careful psychometric work will therefore be essential to examine the factorial structure, reliability, and cross-cultural validity of the proposed questionnaires.

Third, the CEEM and its associated instruments have not yet been empirically tested. As such, the relationships proposed between needs, resources, communication, energy, and engagement should be understood as theoretical propositions rather than established empirical findings. Longitudinal and dyadic studies will be needed to evaluate the model’s assumptions, its sensitivity to change over time, and its relevance across different populations and relational contexts.

## 8 Conclusion

The CEEM provides a new lens on relational experience by integrating energy, engagement, needs, resources, and communication. It complements existing models by offering a falsifiable metric and by opening avenues for innovative empirical research. The CEEM is the first model of romantic relationships to conceptualize the couple as a system for regulating fundamental emotions, and it provides an operational theory of relational burnout. With the development of CEEI and CNQ, it becomes possible to test, validate, and refine the CEEM. In doing so, CEEM furnishes an integrative and operational framework for understanding relational vitality and guiding clinical interventions.

## References

[ref1] Aron A. Aron E. N. Smollan D. (1992). Inclusion of other in the self scale and the structure of interpersonal closeness. J. Pers. Soc. Psychol. 63, 596–612. doi: 10.1037/0022-3514.63.4.596

[ref2] Bernstein D. P. Fink L. Handelsman L. Foote J. (2011). Childhood trauma questionnaire. Washington, DC: APA PshycNET.

[ref3] Bodenmann G. (1995). A systemic-transactional conceptualization of stress and coping in couples. Swiss J. Psychol. 54, 34–59.

[ref4] Bodenmann G. (2005). “Dyadic coping and its significance for marital functioning” in Couples coping with stress: emerging perspectives on dyadic coping. Decade of Behavior (Washington, DC: American Psychological Association).

[ref5] Brennan K. A. Clark C. L. Shaver P. (1998). “Self-report measurement of adult attachment: an integrative overview” in Attachment theory and close relationships. eds. Simpson J. A. Rholes W. S. (New York, NY: Guilford Press), 46–76.

[ref6] Carver C. S. Scheier M. F. Weintraub J. K. (1989). Assessing coping strategies: a theoretically based approach. J. Pers. Soc. Psychol. 56, 267–283. doi: 10.1037//0022-3514.56.2.267, 2926629

[ref7] Cloitre M. Garvert D. W. Brewin C. R. Bryant R. A. Maercker A. (2013). Evidence for proposed ICD-11 PTSD and complex PTSD: a latent profile analysis. Eur. J. Psychotraumatol. 15:4. doi: 10.3402/ejpt.v4i0.20706, 23687563 PMC3656217

[ref8] Davis M. (1980). A multidimensional approach to individual differences in empathy. J. Pers. Soc. Psychol 10:3480.

[ref9] Deci E. L. Ryan R. M. (2000). The ‘what’ and ‘why’ of goal pursuits: human needs and the self-determination of behavior. Psychol. Inq. 11, 227–268. doi: 10.1207/S15327965PLI1104_01

[ref10] Demerouti E. Bakker A. B. Nachreiner F. Schaufeli W. B. (2001). The job demands-resources model of burnout. J. Appl. Psychol. 86, 499–512. doi: 10.1037/0021-9010.86.3.499, 11419809

[ref11] Demerouti E. Bakker A. B. Vardakou I. Kantas A. (2003). The convergent validity of two burnout instruments: a multitrait-multimethod analysis. Eur. J. Psychol. Assess. 19, 12–23. doi: 10.1027//1015-5759.19.1.12

[ref12] Demerouti E. Mostert K. Bakker A. B. (2010). Burnout and work engagement: a thorough investigation of the independency of both constructs. J. Occup. Health Psychol. 15, 209–222. doi: 10.1037/a0019408, 20604629

[ref13] Feeney B. C. Collins N. L. (2015). Thriving through relationships. Curr. Opin. Psychol. 1, 22–28. doi: 10.1016/j.copsyc.2014.11.001, 25774382 PMC4356946

[ref14] Fowers B. J. Olson D. H. (1989). Enrich marital inventory: a discriminant validity and cross-validation assessment. J. Marital. Fam. Ther. 15, 65–79. doi: 10.1111/j.1752-0606.1989.tb00777.x, 21118433

[ref15] Funk J. L. Rogge R. D. (2007). Testing the ruler with item response theory: increasing precision of measurement for relationship satisfaction with the couples satisfaction index. J Fam Psychol 21, 572–583. doi: 10.1037/0893-3200.21.4.572, 18179329

[ref16] Gilbert P. Clarke M. Hempel S. Miles J. N. V. Irons C. (2004). Criticizing and reassuring oneself: an exploration of forms, styles and reasons in female students. Br. J. Clin. Psychol. 43, 31–50. doi: 10.1348/014466504772812959, 15005905

[ref17] Hart O. Nijenhuis E. R. S Steele K. (2007). The haunted self: Structural dissociation and the treatment of chronic traumatization. New York: W. W. Norton & Company.

[ref18] Hatfield E. Rapson R. L. (1990). “Passionate love in intimate relationships” in Affect and social behavior. Studies in emotion and social interaction (Paris: Editions de la Maison des Sciences de l’Homme).

[ref19] Hatfield E. Sprecher S. (1986). Measuring passionate love in intimate relationships. J. Adolesc. 9, 383–410. doi: 10.1016/s0140-1971(86)80043-4, 3805440

[ref20] Hirschfeld R. M. Klerman G. L. Gough H. G. Barrett J. Korchin S. J. Chodoff P. (1977). A measure of interpersonal dependency. J. Pers. Assess. 41, 610–618. doi: 10.1207/s15327752jpa4106_6, 592089

[ref21] Iglesias García M. Urbano Contreras A. Martínez-González R.-A. (2019). Scale of self-perceived communication in the couple relationship (SCCR). An. Psicol. 35, 314–322. doi: 10.6018/analesps.35.2.334451

[ref22] Karandashev V. Evans N. D. Zarubko E. Neto F. Evans M. Artemeva V. . (2020). Physical attraction scale—short version: cross-cultural validation. J. Relationsh. Res. 11:e17. doi: 10.1017/jrr.2020.17

[ref23] Karney B. R. Bradbury T. N. (1995). The longitudinal course of marital quality and stability: a review of theory, methods, and research. Psychol. Bull. 118, 3–34. doi: 10.1037/0033-2909.118.1.3, 7644604

[ref24] Kaufman E. A. Xia M. Fosco G. Yaptangco M. Skidmore C. R. Crowell S. E. (2023). The difficulties in emotion regulation Scale Short Form (DERS-SF): validation and replication in adolescents and adult samples. J Psychopathol Behav Assess. 38, 443–455. doi: 10.1037/t89267-000 PMC12788809 41522882

[ref25] Kenny D. A. Kashy D. A. Cook W. L. (2006). Dyadic data analysis. New York, NY: The Guilford Press.

[ref26] Michael K. Ben-Zur H. (2024). Couples’ psychological resources and marital satisfaction: the mediating role of marital support. Eur. J. Psychol. 20, 303–316. doi: 10.5964/ejop.11769 39678301 PMC11636713

[ref27] Panksepp J. (1998). Affective neuroscience: The foundations of human and animal emotions. Oxford: Oxford University Press.

[ref28] Penke L. Asendorpf J. B. (2008). Beyond global sociosexual orientations: a more differentiated look at sociosexuality and its effects on courtship and romantic relationships. J. Pers. Soc. Psychol. 95, 1113–1135. doi: 10.1037/0022-3514.95.5.1113, 18954197

[ref29] Pines A. M. (1996). Couple burnout: causes and cures. London: Taylor & Frances/Routledge.

[ref30] Pines A. M. Renato N. (2003). The relationship between career and couple burnout: implications for career and couple counseling. J. Employ. Couns. 40, 50–64. doi: 10.1002/j.2161-1920.2003.tb00856.x

[ref31] Ross J. M. Nguyen T. P. Karney B. R. Bradbury T. N. (2022). Three tests of the vulnerability-stress-adaptation model: independent prediction, mediation, and generalizability. Front. Psychol. 13:921485. doi: 10.3389/fpsyg.2022.921485, 35967721 PMC9366884

[ref32] Rubin Z. (1970). Measurement of romantic love. J. Pers. Soc. Psychol. 16, 265–273. doi: 10.1037/h0029841, 5479131

[ref33] Sandberg J. G. Busby D. M. Johnson S. M. Yoshida K. (2012). The brief accessibility, responsiveness, and engagement (BARE) scale: a tool for measuring attachment behavior in couple relationships. Fam. Process 51, 512–526. doi: 10.1111/j.1545-5300.2012.01422.x, 23230982

[ref34] Selye H. (1956). The stress of life. New York, NY: McGraw-Hill.

[ref35] Singer J. Labunko B. Alea N. Baddeley J. (2015). Mutuality and the marital engagement – Type of union scale [ME (to US)]: Empirical support for a clinical instrument in couples therapy, in eds. K. Skerrett, and K. Fergus. Couple Resilience, 1, 123–138. doi: 10.1007/978-94-017-9909-6_7

[ref36] Spanier G. B. (1976). Measuring dyadic adjustment: new scales for assessing the quality of marriage and similar dyads. J. Marriage Fam. 38, 15–28. doi: 10.2307/350547

[ref37] Straus M. A. Hamby S. L. Boney-McCoy S. Sugarman D. B. (1996). The revised conflict tactics scales (CTS2): development and preliminary psychometric data. J. Fam. Issues 17, 283–316. doi: 10.1177/019251396017003001

[ref38] Thompson A. E. Theis R. Willhite R. Dębska J. (2025). Love on empty: the development and validation of a comprehensive scale to measure burnout in modern relationships. Behav. Sci. 15:1737. doi: 10.3390/bs15121737, 41464080 PMC12729541

[ref39] Weinryb R. M. Barber J. P. Foltz C. Göransson S. G. M. Gustavsson J. P. (2000). The central relationship questionnaire (CRQ): psychometric properties in a Swedish sample and cross-cultural studies. J. Psychother. Pract. Res. 9, 201–212, 11069133 PMC3330609

[ref40] Yela C. (2006). The evaluation of love simplified version of the scales for Yela’s Tetrangular model based on Sternberg’s model. Eur. J. Psychol. Assess. 22, 21–27. doi: 10.1027/1015-5759.22.1.21

[ref41] Yerkes R. M. Dodson J. D. (1908). The relation of strength of stimulus to rapidity of habit formation. J. Comp. Neurol. Psychol 18, 459–482. doi: 10.1002/cne.920180503

